# Integration of a multimeric STAT consensus site into the adenoviral genome to interfere with IFN signaling represents a novel concept to improve oncolytic virotherapy

**DOI:** 10.1128/jvi.01068-26

**Published:** 2026-08-10

**Authors:** Dongbiao Wang, Sonja Nassua, Klaus Mantwill, Yuling Zhao, Jacqueline C. Bambach, Jürgen E. Gschwend, Per Sonne Holm, Roman Nawroth

**Affiliations:** 1 Department of Urology, TUM Klinikum Rechts der Isar, Technical University of Munich 27190, Munich, Germany; 2 Department of Oral and Maxillofacial Surgery, Innsbruck Medical University 84949, Innsbruck, Austria; Tufts University, Boston, Massachusetts, USA

**Keywords:** decoy oligonucleotide, adenovirus, oncolytic virotherapy, JAK/STAT, antiviral interferon response

## Abstract

Adenovirus infection triggers an interferon (IFN) response that reduces replication of the viral genome early in the adenoviral life cycle. In oncolytic virus therapy, viral replication is essential for effective cell lysis, which subsequently can activate a systemic immune response against the tumor. We show that different members of the JAK/STAT protein family redundantly contribute to the suppression of adenoviral replication in bladder cancer-derived cells. This mechanism requires the expression of E1A; however, it does not affect expression levels of E1A, but rather its transactivation of E1B55K, and particularly E4. We developed a novel concept to sequester STAT isoforms from host cells by using a multimeric STAT consensus sequence that was introduced into the E3 region of the oncolytic virus XVir-N-31, resulting in XVir-N-14. This virus exhibits a higher potency in replication, cell lysis, and particle formation than XVir-N-31. This approach of sequestering DNA-binding molecules to the viral genome might offer an alternative to combination therapy with JAK/STAT inhibitors, which would not only suppress the desired systemic immune response but also cause serious adverse effects.

**IMPORTANCE:**

Successful application of oncolytic virotherapy requires efficient virus replication that can be suppressed by the host’s cell antiviral IFN response. We extend in this study the insight into the underlying molecular mechanisms in bladder cancer cells. Suppression of JAK/STAT activity induces an E1A-dependent upregulation of early gene expression, although the E1A expression level is not affected. The effect is not observed when overexpressing E1B55K or E4orf6 in the viral genome, indicating a regulatory role of STAT molecules on the transactivation capacity of E1A. We developed a novel concept to interfere with the JAK/STAT signaling pathway by cloning a multimeric STAT consensus sequence in the E3 region of the oncolytic virus XVir-N-31. The resulting virus XVir-N-14 shows improved replication, cell lysis, and particle formation. Trapping a molecular suppressor to the viral genome supplements previous strategies to modify the host’s immune response by cloning shRNAs or molecular inhibitors against the JAK/STAT signaling pathway.

## INTRODUCTION

Oncolytic virotherapy is an emerging immunotherapy for cancer treatment, and recent clinical trial data have highlighted the effectiveness and safety of using an oncolytic adenovirus for treating bladder cancer (1–3). One challenge in optimizing an oncolytic virus (OV) for therapy is controlling the host’s antiviral immune response during replication. This response is predominantly regulated by interferon (IFN) signaling, which subsequently activates the JAK/STAT signaling pathway (4–6).

Initial recognition of an adenoviral infection is mediated by toll-like receptors or the cGAS/STING DNA-sensing complex, a pathogen recognition receptor that activates the interferon response. Interferons are classified into three types: I (e.g., IFN-α, IFN-β), II (IFN-γ), and III (IFN-λ), and are important mediators of the adaptive and innate immune response (7). Interferons activate the canonical JAK/STAT signaling cascade through cytokine receptor-associated Janus kinases (JAK1–3 and TYK2). Activation results in the autophosphorylation of JAKs, creating docking sites for members of the signal transducers and activators of transcription (STAT) family, which are subsequently tyrosine phosphorylated. This process leads to STAT dimerization, nuclear translocation, DNA binding, and transcriptional control of multiple interferon-stimulated genes (ISGs) (8, 9).

The STAT protein family comprises seven members: STAT1–4, STAT5a, b, and STAT6 (10). Several virus families exploit STAT proteins to facilitate viral gene expression and viral DNA replication, such as Epstein-Barr virus (EBV), Kaposi’s sarcoma herpesvirus (KSHV), and human immunodeficiency virus type 1 (HIV-1) (6). In contrast, C-terminally truncated STAT5 can suppress HIV-1 gene expression (11).

Although for adenoviruses, no direct regulatory function of STAT proteins on promoter activation has been shown to date, IFN signaling was demonstrated to affect the adenoviral transcriptional control of the viral E1A enhancer by reducing GABP-mediated transactivation and by controlling E2F release from Rb1. For this IFN-dependent downregulation, the second, 3′ located E2F-binding site was identified as functionally important (12, 13).

E1A also participates in the multilayered suppression of host immunity by adenoviruses (14). It directly blocks expression of ISGs by interfering with the p300/CBP protein complex or by regulating histone 2B mono-ubiquitination through the ubiquitin ligase RNF20/hBRE1 (15, 16, 17). It can also directly bind STAT1 and thereby inhibit IFN-induced expression of target genes, such as transcripts for ICAM-1 and IRF-1 (18). Interestingly, following wild-type adenovirus infection, phosphorylated STAT1 has been observed to accumulate at viral replication centers (19). The authors interpreted these data as an evolutionary adaptation that limits STAT1 access to its cellular target genes.

Another important protein encoded in the E1 region is E1B55K, which has been shown to suppress transcription of IFN-inducible genes in conjunction with p53 (20, 21). Moreover, the protein complex of E1B55K and E4orf3, together with cellular factors, blocks antiviral IFN responses by rearrangement of the promyelocytic leukemia (PML) protein complex and proteasome-mediated repression of the Mre11-Rad50-Nbs1 (MRN) complex and ISGs such as the death-domain-associated (Daxx) defense factor (22–24, 25).

Despite these viral strategies to counteract the host’s immune defense, IFN stimulation was shown to reduce adenoviral replication (12). Consequently, targeting the JAK/STAT pathway with small-molecule inhibitors or by knocking down JAK or STAT proteins can improve adenoviral replication (19, 26–28). These findings highlight the critical role of the JAK/STAT pathway in modulating host–virus interactions and thus also make it a potential target for optimizing oncolytic virotherapy.

We aimed to better understand the mechanism by which the JAK/STAT pathway interferes with adenoviral replication to develop novel strategies to improve the replication and oncolytic efficacy of the oncolytic adenovirus XVir-N-31. XVir-N-31 is characterized by deletions of E1A13S, E1B19K, and in the E3 region, which results in conditional replication in cancer cells that express Y-box protein 1 (YB-1) (29). However, the absence of E1A13S delays replication compared with the wild-type adenovirus, as it acts as a dominant factor in the transactivation of viral early gene expression (30, 31). We show that IFN stimulation of bladder cancer-derived cell lines results in substantial inhibition of adenoviral replication. Therefore, we analyzed in detail the effects of JAK1/2 and STAT inhibitors, as well as siRNAs directed against STAT1, STAT3, and STAT5, on XVir-N-31 compared to a wild-type adenovirus derivative (Ad5-dE3-RGD). All these strategies synergistically improved cell lysis, replication, and particle formation across different bladder cancer cell lines. Our data also indicate a redundancy among the different members of the JAK/STAT protein family. We also investigated changes in viral early gene and protein expression following JAK1/2 inhibition and identified increased expression of E1B55K, E2A, and E4 isoforms, but no change in E1A expression. Importantly, the effect of JAK1/2 inhibition was completely abolished in E1-/E1A-deleted adenoviruses, indicating that E1A is an essential prerequisite for the observed effects. Overexpressing E1B55K and/or E4orf6 in genetically engineered adenoviruses also eliminated the effect of JAK inhibition and revealed E4orf6 as the predominant gene contributing to this effect. Based on these results, we developed a novel strategy for the design of oncolytic virotherapy by introducing a multimer of a STAT consensus sequence into the viral genome, trapping nuclear STAT molecules at this site. This novel oncolytic adenovirus (XVir-N-14) exhibited enhanced replication and cell lysis compared to the parental XVir-N-31, thereby validating this concept for optimizing oncolytic virotherapy.

## MATERIALS AND METHODS

### Cells

Cell lines T24 (ATCC HTB-4), 5637 (ATCC HTB-9), and HEK293 were purchased from ATCC. UM-UC-3 cells were obtained from Professor WA Schulz, Düsseldorf, Germany. T24shRb cells were established from the cell line T24 (ATCC HTB-4) as described previously (32). Cells were grown in Dulbecco’s modified Eagle’s medium (DMEM, Sigma-Aldrich) supplemented with 10% fetal bovine serum and 1% penicillin-streptomycin, and maintained at 10% CO2.

### Small molecule inhibitor treatment

Inhibitors Ruxolitinib (#INCB18424), Itacitinib (#INCB39110), and Napabucasin (#BBI608) were obtained from Selleck Chemicals and dissolved in DMSO.

Palbociclib (#PD-0332991, Sigma-Aldrich) was dissolved in ddH20. Working concentrations were prepared freshly for immediate use. For inhibitors dissolved in DMSO, the highest DMSO concentration used in the assays served as a control.

### Adenovirus and infection

The 10xSTAT trapping sequence was synthesized by Thermo Scientific (5′AAACGGATCCCATTTCCAGGAAATCCATTTCCAGGAAATCCATTTCCAGGAAATCCATTTCCAGGAAATCCATTTCCAGGAAATCCATTTCCAGGAAATCCATTTCCAGGAAATCCATTTCCAGGAAATCCATTTCCAGGAAATCCATTTCCAGGAAATCAGATCTCCAGCGCTCGAGGTT-3′). It was provided in the vector pMA-RQ-10xSTAT and cloned into the deleted E3 region of XVir-N-31, resulting in the novel virus XVir-N-14.

To enable overexpression of E1B55K and E4orf6, CMV promoter sequences were fused to their respective cDNAs and cloned into the E3 region of XVir-N-31, resulting in XVir-N-5 and XVir-N-6, respectively.

Adenoviruses were produced in HEK293 cells and purified by CsCl-gradient centrifugation as previously described (33), followed by size-exclusion (PD-10 Desalting Columns, GE Healthcare, Freiburg, Germany) and anion exchange chromatography (ÄKTA pure chromatography system, HiTrap Q XL Column, Cytiva).

### Infection

Cells were seeded in 10 cm dishes or 6- or 12-well plates and pretreated or transfected 24 h later, as described in the results section. Infection was performed 24 h after seeding, unless otherwise stated, with the described multiplicity of infection (MOI) in serum-free DMEM for 1 h. Afterward, complete medium was added, with or without inhibitor treatment.

### Cytotoxicity assay

To determine cell viability, 2 × 10^4^ cells were seeded into 12-well plates and treated and infected as described. After 5 days, cells were fixed with 10% ice-cold trichloroacetic acid at 4°C for 1 h and stained using a 1% acetic acid solution containing 0.05% sulforhodamine B (SRB, #S9012, Sigma-Aldrich) for 30 min. To quantify cell survival, membrane-bound SRB was dissolved in 10 mM Tris buffer (pH 10), and the absorbance was measured at 590 nm. Synergistic effects on cell viability were evaluated using the “Zero interaction potency” (ZIP) model by Synergy Finder (34).

### Viral replication and particle determination

Cells seeded in 6-well plates (1 × 10^5^ cells/well) were treated 24 h later with inhibitors. Infection was performed 1 day post-treatment as described above. For viral replication analysis, infected cells were lysed at the desired time points using a buffer containing 0.5% SDS, 100 mM NaCl, 10 mM Tris-HCl pH 8, 25 mM EDTA, pH 8, and ddH2O.

DNA was then extracted using Phenol–Chloroform–Isoamyl Alcohol (Sigma-Aldrich, #P3803). Viral replication was assessed by quantifying adenoviral fiber DNA using the GoTaq qPCR Master Mix (#A6010, Promega) for real-time qPCR on a Bio-Rad CFX96 Touch Real-time PCR System. Cellular actin served as a reference for viral DNA copies, and data were analyzed using the ∆∆Ct method (35, 36).

Viral particle formation was performed by collecting cell lysates from infected cells at 72 h post-infection (hpi) and performing a hexon titer test using HEK293 cells, as previously described (36).

### Plasmid and siRNA transfection

Transfection of the pMA-RQ-10xSTAT plasmid (Thermo Scientific) was performed using FuGENE HD transfection reagent (Promega, #E2311) following the manufacturer’s instructions. For siPool-mediated knockdowns, reverse transfection was performed according to the siTOOLs Biotech protocol using Lipofectamine RNAiMAX (Thermo Scientific, #13778). Cells (1 × 10^5^ cells/well) were transfected in 6-well plates, using a transfection solution with a final concentration of 1 nM of the siCtrl/siSTAT1/siSTAT3/siSTAT5 pools (#si-G020-799, #si-G020-6772, #si-G020-6774, and #si-G020-6776; siTOOLs Biotech). To analyze siPool efficacy, protein samples were collected 24 and 48 h after transfection and analyzed by western blot. Inhibitor treatment was applied 24 h post-transfection (hpt), and infection was performed the following day.

### Gene expression analysis

For gene expression analysis, cells (1 × 10^5^ cells/well) were seeded in 6-well plates and infected as described above. Cells were harvested at the indicated time points, and RNA was extracted using phenol:chloroform (Ambion, #AM9720) and reverse transcribed (High-Capacity cDNA reverse transcription kit, Thermo Fisher, #4368814) using 1 µg RNA. The cDNA was quantified using real-time qPCR (Bio-Rad CFX96 Touch Real-time PCR System). Primers used to quantify viral genes were obtained from Eurofins (36). Relative quantification was analyzed using the ∆∆Ct method, and viral genes were normalized to cellular β-actin cDNA levels.

### Immunoblot analysis

Immunoblot analysis was performed as described before, using the primary antibodies E1A (Sc-25; SantaCruz), E2A, E1B55K, E4orf6 (B6-8, 2A6, and RS-A31; Helmholtz-Institute Munich), Hexon (ABIN2686029, Antibodies Online), STAT1, STAT2(D9J7L), STAT3(124H6), STAT4 (C46B10), STAT5 (D2O6Y), STAT6 (D3H4), p-STAT1 (58D6), p-STAT2, p-STAT3 (D3A7), p-STAT4, p-STAT5, p-STAT6, GAPDH (14C10) (#9172, #724604, #9139, #2653, #94205, #94205, #9167, #4441, #9145, #5267, #9351, #9361, #2118; Cell Signaling Technology), and the secondary peroxidase-conjugated anti-mouse IgG or anti-rabbit IgG (#715-036-150, #711-036-152; Dianova).

### DNA pull-down assay

6 × 10^6^ cells were seeded in a 15 cm dish and harvested at 80% confluency 24 h later using RIPA buffer (25 mM Tris, pH 7.4, 150 mM NaCl, 1% Igepal, 0.5% sodium deoxycholate, 0.1% SDS, and protease inhibitor). After centrifugation for 30 min at 31,000 × *g* (5427 R, Eppendorf), protein concentration was determined by Pierce BCA Protein Assay (Thermo Scientific, #23225), and samples were adjusted to a concentration of 500 µg/mL using the binding buffer (20 mM Hepes, pH 7.4, 100 mM KCl, 1.5 mM MgCl2, 0.5 mM EDTA, 20% glycerol, and 1 mM DTT). The following 5′ biotin-labeled and -unlabeled oligonucleotides (Metabion) M67: CATTTCCCGTAAATCAT (37), 1xSTAT: ATCCCATTTCCAGGAAATCC, and 4xSTAT: ATCCCATTTCCAGGAAATCCATTT CCAGGAAATCCATTTCCAGGAAATCCATTTCCAGGAAATCCAT were annealed using a concentration of 0.5 µM in TE buffer (94°C for 2 min, 70°C for 5 min, 60°C for 1 min, 50°C for 1 min, 40°C for 1 min, 30°C for 1 min, and 20°C for 1 min). Probes were prepared by adding 20 µL of the annealed oligonucleotides to 500 µL of the adjusted whole-cell lysate. An additional 20 µL of the annealed unlabeled oligonucleotides was added as a negative control. After a 1-h incubation rotating at room temperature (RT), 20 µL high-capacity streptavidin agarose resin (#20361; Thermo Scientific) was added, and samples were incubated for another hour rotating at RT. Finally, samples were centrifuged at 5,000 × *g* for 1 min (5427 R, Eppendorf) and washed three times with 1 mL washing buffer (25 mM Tris, pH 7.4, 15 mM NaCl, 1% NP40, and 0.5% sodium deoxycholate). Forty microliters of 4× SDS-PAGE buffer was added, and the samples were heated at 95°C for 5 min. For the subsequent detection of binding proteins, immunoblot analysis was performed as described above.

### ChIP analysis

For ChIP analysis, 4 × 10^6^ cells were seeded in a 10 cm plate, infected with XVir-N-14 (MOI 50), and harvested 18 h after infection. ChIP was performed according to the protocol described by Rodríguez-Ubreva et al. (38). After crosslinking for 10 min using a concentration of 1% formaldehyde and subsequent 5-min incubation with glycine (0.1 M), cells were incubated for 30 min in detergent lysis buffer (1% SDS, 10 mM EDTA, 50 mM Tris/HCl, pH 8.1, protease inhibitor) and subsequently sonicated for 30 s, 9 cycles (Sonoplus Serie HD 2000, Bandelin). Immunoprecipitation was performed using the STAT1 antibody #9172 from Cell Signaling Technology and Pierce Protein A/G Plus Agarose Beads (#20423; Thermo Scientific). The immunoprecipitated DNA was isolated using the Monarch PCR Clean-up Kit from NEB and quantified by qPCR to amplify the E3 region using the primers (Omnilab, 5′-CAAGGAGGTGCTGCTGAATA-3′, 5′-GTAGCCTGATTCGGGAGTTTAC-3′).

## RESULTS

### Effect of JAK1/2 inhibition on oncolytic adenoviral therapy in bladder cancer cells

The aim of this study was to gain a mechanistic understanding of the effects of IFN signaling on adenovirus replication to develop strategies to enhance the therapeutic efficacy of oncolytic adenoviruses. Using bladder cancer cell lines as a model system, we investigated the oncolytic adenovirus XVir-N-31 (formerly Ad-delo3-RGD) as well as the type 5 adenovirus, Ad5-dE3-RGD, which served as a wild-type derivative control (29).

**Fig 1:**
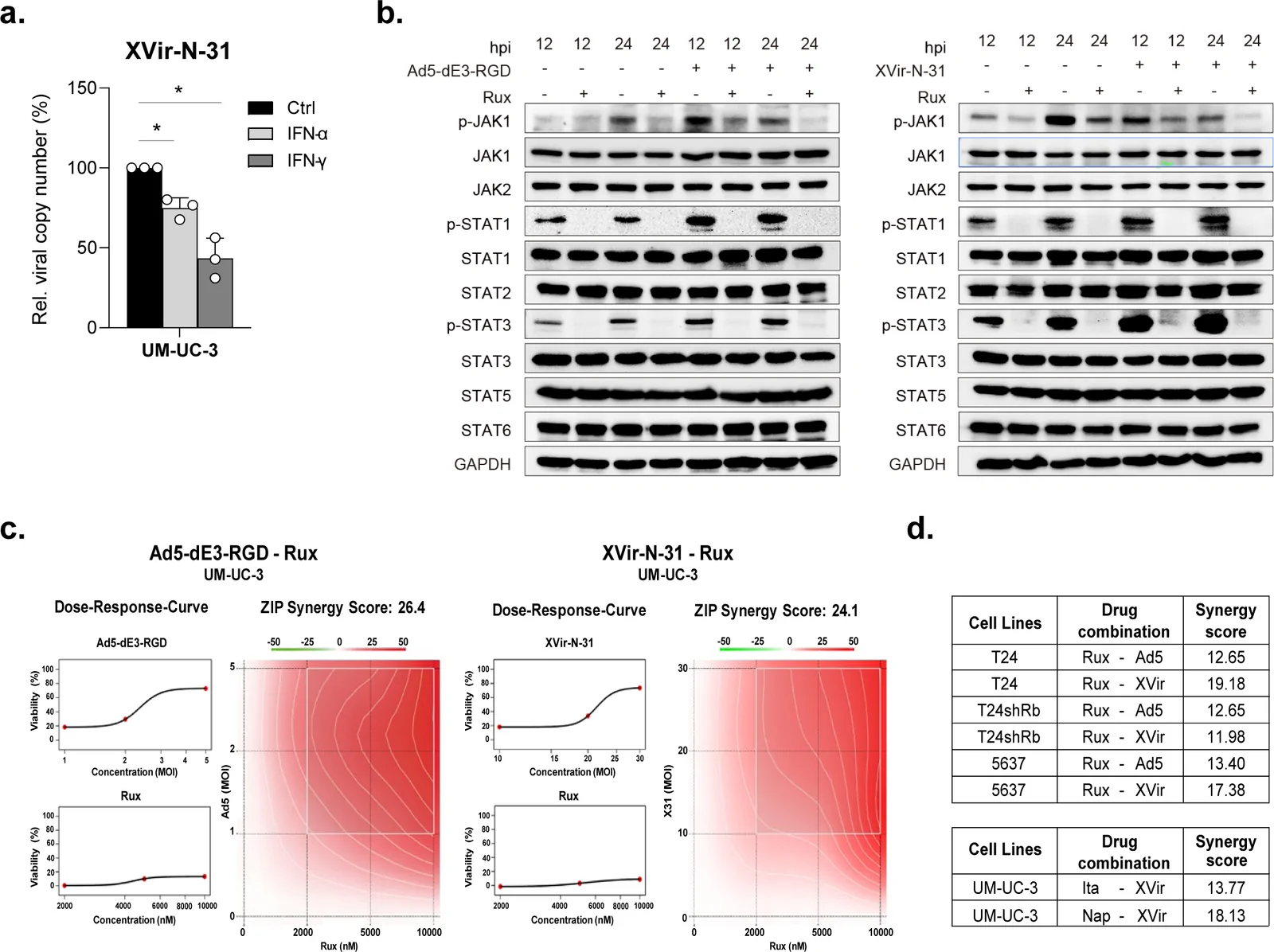
JAK/STAT inhibition increases virus-induced cell killing. (**a**) UM-UC-3 cells were infected with XVir-N-31 (MOI 10) and treated with 50 pM IFN-α or 25 pM IFN-γ. Viral copy numbers were analyzed at 24 hpi by quantifying viral fiber DNA and compared to the untreated control (*n* = 3, mean ± SE). Data were analyzed by an unpaired two-tailed *t*-test (*, *P* ≤ 0.05). (**b**) Protein expression was analyzed at the indicated time point by western blotting of UM-UC-3 cells pretreated without Ruxolitinib for 24 h and infected with Ad5-dE3-RGD (MOI 1) or XVir-N-31 (MOI 10) (*n* = 3, one representative blot is shown). (**c and d**) Bladder cancer cell lines (UM-UC-3, 5637, T24, and T24shRb) were pretreated with JAK1/2 inhibitors (Ruxolitinib 10 µM [Rux], Itacitabine 10 µM [Ita], Napabucasin 0.2 µM [Nap]) for 24 h and then infected with the indicated MOIs of Ad5-dE3-RGD (Ad5) or XVir-N-31 (XVir). Cell viability was assessed at 5 dpi (*n* = 3, mean ± SE). Synergy scores between JAK1/2 inhibitors and viruses across different cell lines were calculated using the ZIP model.

To verify the reported effects of interferon signaling on adenoviral replication, we first analyzed the impact of IFN stimulation on XVir-N-31 replication in the bladder cancer cell line UM-UC-3. Treatment of infected cells with IFN-α or IFN-γ reduced viral replication by approximately 30% and 50%, respectively (Fig. 1a).

Next, we examined the mechanistic effect of an adenoviral infection on the JAK/STAT signaling pathway in UM-UC-3 cells, analyzing the expression level and phosphorylation status of JAK1 and -2 and STAT1-6 with or without adenovirus infection at 12 and 24 hpi. Expression of JAK-1/-2 and all STAT isoforms, except for STAT4, was detected (Fig. 1b). Constitutive phosphorylation of JAK-1, STAT-1, and -3 was observed at both time points, with levels slightly higher at 24 h.

Upon virus infection, using Ad5-dE3-RGD and XVir-N-31, no change in expression levels was observed, but phosphorylation of JAK-1, STAT-1, and STAT-3 was upregulated. As for JAK-1, phosphorylation was highest at 12 hpi with both viruses but decreased almost to the level of uninfected cells at 24 h. Furthermore, JAK1/2 inhibition prevented phosphorylation of JAK-1, STAT-1, and -3 after infection.

It has been demonstrated that inhibition of specific members of the JAK/STAT signaling pathway improves adenoviral replication (19, 26–28). We confirmed and extended these results using different small-molecule inhibitors against JAK and STAT proteins and combined them with Ad5-dE3-RGD and XVir-N-31 in human bladder cancer cells. Cell viability was analyzed using a Sulforhodamine B assay at 5 days post-infection (dpi). We first tested the specific JAK1/2 inhibitor Ruxolitinib in the four cell lines UM-UC-3, T24, the Rb1-negative cell line 5637, and T24shRb, a cell line stably expressing an Rb1 shRNA. The interaction patterns between Ruxolitinib and viruses were analyzed using the ZIP model in SynergyFinder 2.0 (34). In this bioanalytic tool, a synergy score larger than 10 indicates synergism between two drugs. Cells were pretreated with increasing concentrations of Ruxolitinib for 24 h, followed by infection with increasing MOIs of Ad5-dE3-RGD or XVir-N-31, respectively (Fig. 1c and d; Fig. S1). While Ruxolitinib as monotherapy showed minor effects, cell viability in all four cell lines was reduced with increasing MOIs of both Ad5-dE3-RGD and XVir-N-31. In combination, virus-induced cell death by both viruses was enhanced up to 30%–65% compared to monotherapy observed at a concentration of 10 µM Ruxolitinib. The combination of Ruxolitinib and Ad5-dE3-RGD or XVir-N-31 induced synergistic effects in all four cell lines, irrespective of their Rb status. When directly comparing the T24 parental cells with the T24shRb cells, no difference in the extent of the response was observed. We additionally used Itacitinib, a selective JAK1 inhibitor, and Napabucasin, a STAT3 inhibitor. These were combined with the oncolytic adenovirus XVir-N-31, yielding results similar to those observed with Ruxolitinib (Fig. 1d; Fig. S1). This finding suggests that, in cancer cells, the effect of JAK/STAT inhibition on virus-induced cell death is not regulated via the Rb/E2F pathway.

### Inhibition of JAK/STAT signaling provokes a rapid, direct increase in viral replication and particle formation

**Fig 2:**
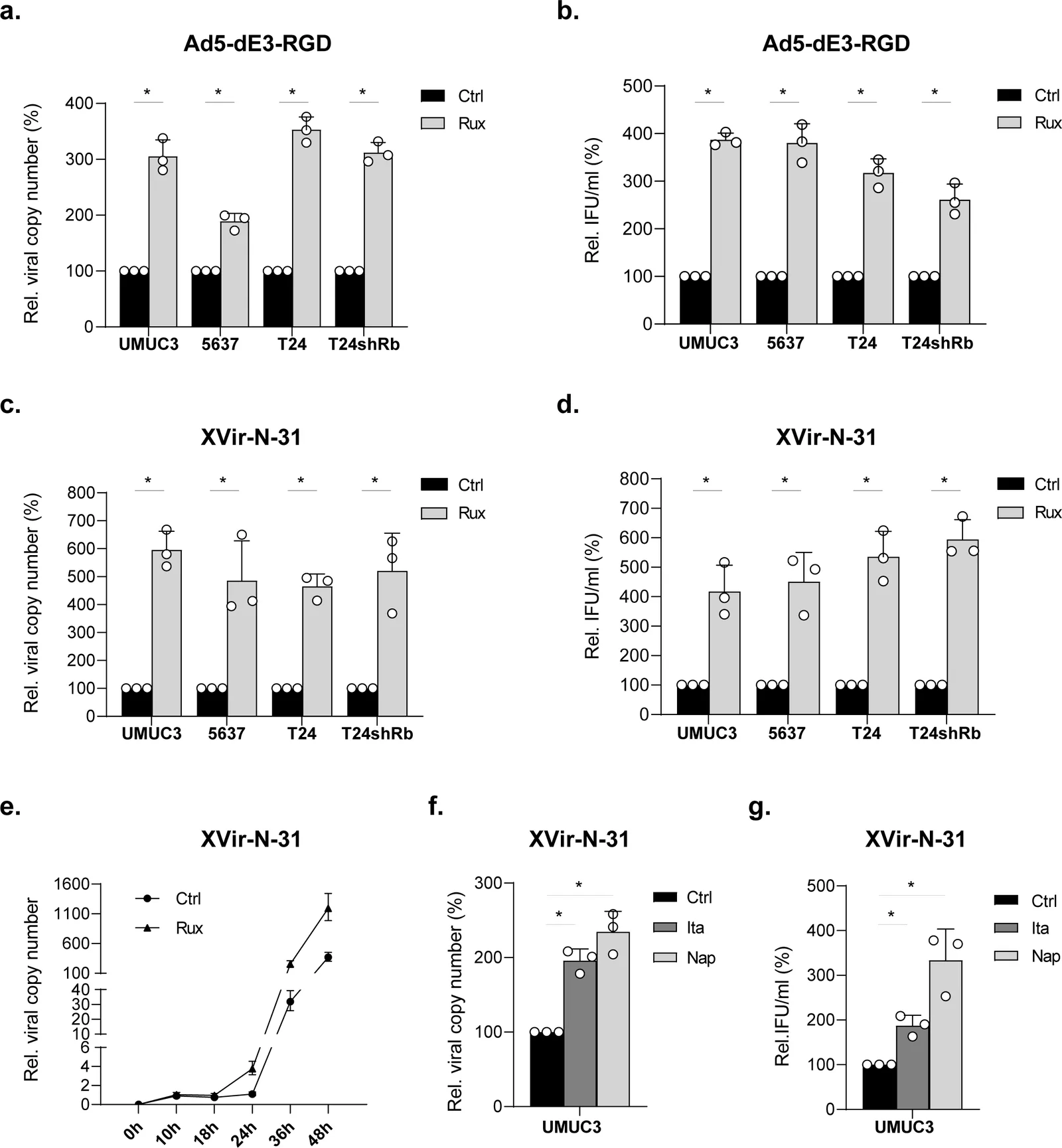
JAK/STAT inhibition increases viral replication. (**a and c**) Cells were pre-treated with Ruxolitinib (10 μM) for 24 h and then infected with the indicated MOI of Ad5-dE3-RGD (UM-UC-3, MOI 1; 5637, MOI 10; T24, MOI 10; and T24shRb, MOI 25) or XVir-N-31 (UM-UC-3, MOI 10; 5637, MOI 20; T24, MOI 25; and T24shRb, MOI 25), respectively. Replication was assessed by qPCR amplification of viral fiber DNA and was compared with the untreated control (*n* = 3, mean ± SE). Data were analyzed using multiple *t*-tests corrected for multiple comparisons by the Holm-Sidak method (*, *P*≤0.05). (**b and d**) Quantification of viral particles was performed by pre-treating cells with Ruxolitinib (10 µM) for 24 h, followed by infection with Ad5-dE3-RGD or at XVir-N-31 of the same MOI, as described in panels **a and c**. The Hexon titer test was performed to determine the infectious units per milliliter (IFU/mL). Data are presented as fold change relative to untreated control (*n* = 3, mean ± SE) and analyzed by using multiple *t*-tests corrected for multiple comparisons by the Holm-Sidak method (*, *P*≤0.05). (**e**) UM-UC-3 cells were pre-treated with Ruxolitinib (10 µM) for 24 h and then infected with XVir-N-31 MOI 10. Cells were collected at the indicated time points, and replication was assessed by qPCR amplifying viral fiber DNA; Data are normalized to the 4 h fiber DNA value (*n* = 3, mean ± SE). (**f and g**) Cells were pre-treated with Itacitinib (10 μM) or Napabucasin (0.2 µM) for 24 h. Next, cells were infected with XVir-N-31 (MOI 10). Replication was measured by qPCR amplification of viral fiber DNA and was compared with the untreated control (*n* = 3, mean ± SE) (**f**). Particle formation was assessed by immunostaining against hexon to determine the infectious units per milliliter (IFU/mL). Data are presented as fold change relative to untreated control. (**g**) (*n* = 3, mean ± SE) and analyzed by using an unpaired two-tailed *t*-test (*, *P* ≤ 0.05).

Treatment with Ruxolitinib significantly increased replication and particle formation of Ad5-dE3-RGD twofold to threefold and of XVir-N-31 approximately fourfold to sixfold (Fig. 2a through d). Treatment with Itacitinib and Napabucasin resulted in similar effects on XVir-N-31 replication and particle formation (Fig. 2f and g). In conclusion, inhibition of the IFN pathway by targeting JAK1/2 improves the replication, particle formation, and cell-killing efficacy of adenovirus type 5 derivatives.

The JAK/STAT pathway regulates numerous downstream effectors that play a role in cellular antiviral responses and cell cycle progression (4, 39, 40). We also asked whether it is necessary to first establish a specific molecular environment in the host cell before infection and tested different pre-treatment regimens of Ruxolitinib and subsequent infection of XVir-N-31 at different MOIs in UM-UC-3 and T24.

No difference in replication or cell viability was observed between the conditions used, indicating that inhibition of the JAK/STAT signaling pathway affects replication immediately and, consequently, the viral life cycle (Fig. S2c through f).

### JAK1/2 inhibition enhances viral gene expression

**Fig 3:**
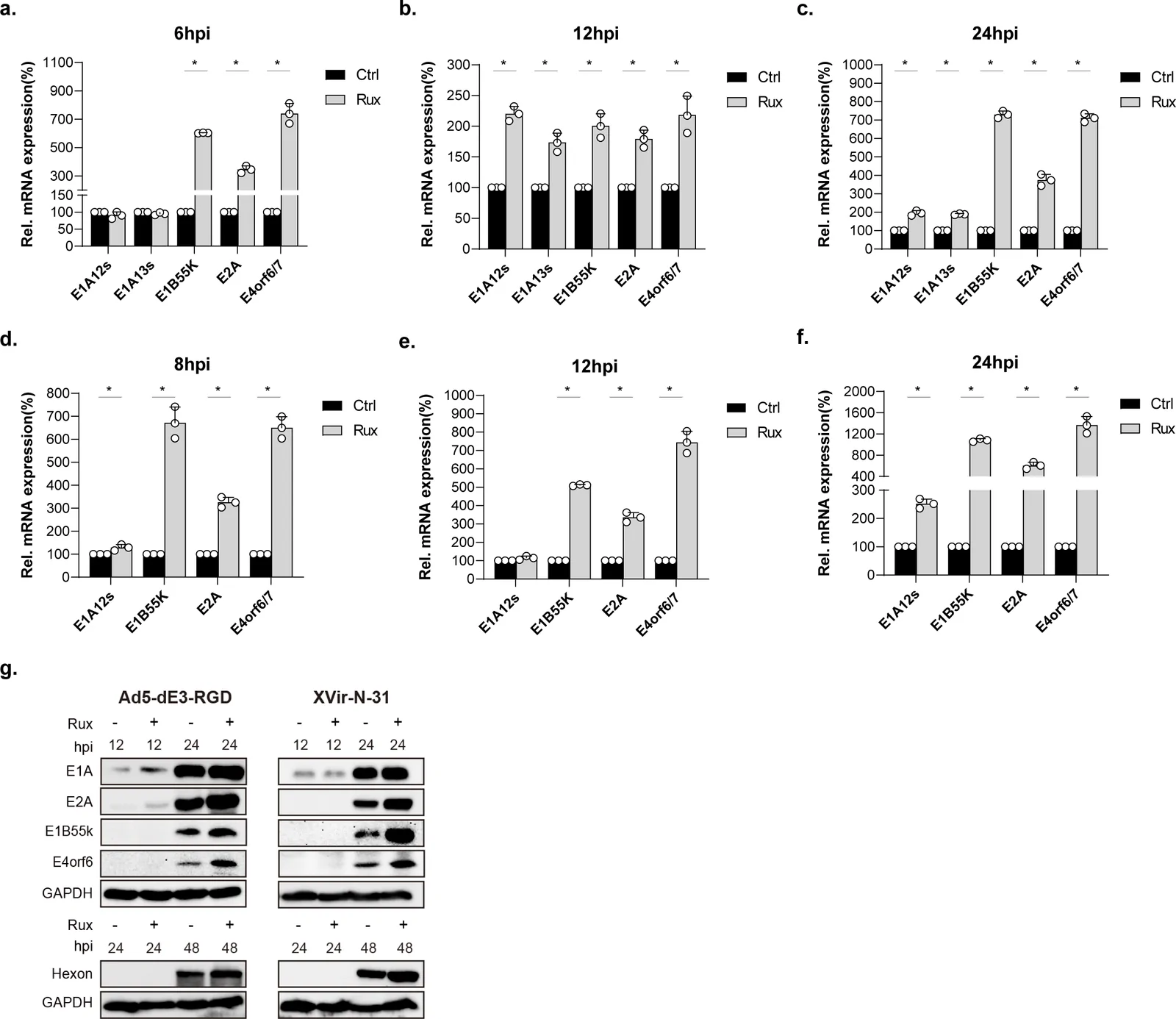
JAK1/2 inhibition impacts viral gene and protein expression at early stages after infection. (**a–f**) Gene expression for viral genes was analyzed in UM-UC-3 cells pre-treated with Ruxolitinib at 10 µM and infected with Ad5-dE3-RGD (MOI 1) or XVir-N-31 (MOI 10). RNA was extracted at the indicated time points, transcribed to cDNA, and viral transcripts were quantified by qPCR. Data are presented relative to untreated control (*n* = 3, mean ± SE) and analyzed by using multiple *t*-tests corrected for multiple comparisons by the Holm-Sidak method (*, *P* ≤ 0.05). (**g**) Viral protein expression was analyzed at the indicated time point by western blotting using lysates of UM-UC-3 cells pretreated with or without Ruxolitinib for 24 h and infected with Ad5-dE3-RGD (MOI 1) or XVir-N-31 (MOI 10). One representative blot is shown in three independent experiments.

To further elucidate the impact of JAK1/2 signaling on adenoviral replication, we performed a time-course analysis of adenoviral gene expression specifically targeting E1A, E1B55K, E2A, and E4 from 6 to 24 hpi. Because the lack of E1A13S in XVir-N-31 causes delayed replication kinetics compared to Ad5-dE3-RGD, the initial time points were defined as 6 hpi for Ad5-dE3-RGD and 8 hpi for XVir-N-31 (30). Ruxolitinib-mediated JAK inhibition robustly upregulated the mRNA transcripts of the early genes E1B55K, E2A and E4orf6/7, at early stages of infection in both viruses (Fig. 3a through f). In contrast, the induction of E1A12S and/or 13S expression was delayed, showing enhancement only from 12 hpi onwards. Furthermore, at later time points, E1A expression exhibited only a modest 2–2.5 increase in both viral strains, which was significantly less pronounced than the upregulation observed for the other viral genes (Fig. 3a and b).

In the next step, we investigated whether changes in transcript levels also affect viral protein levels. UM-UC-3 cells were infected with Ad5-dE3-RGD and XVir-N-31, and protein lysates were harvested at 12 and 24 hpi. Ruxolitinib-mediated JAK inhibition resulted in a generalized upregulation of adenoviral E1B55K, E2A, E4orf6, and Hexon protein levels in both viruses at 24 hpi. E1A expression showed a slight increase 12 h after infection in cells treated with Ruxolitinib and infected with Ad5-dE3-RGD, whereas it remained unchanged in cells infected with XVir-N-31.

Also, at 24 h, the effect of Ruxolitinib on increased E1A expression was modest in both viruses, especially when compared to the effect on E1B55K, E2A, E4orf6, and Hexon (Fig. 3g). These findings are consistent with our transcriptional findings, suggesting that JAK/STAT inhibition enhances E1A expression exclusively during the later stages of viral replication, while leaving baseline E1A protein levels unaffected at early time points.

### The effect of JAK1/2 inhibition on viral replication requires expression of E1A

**Fig 4:**
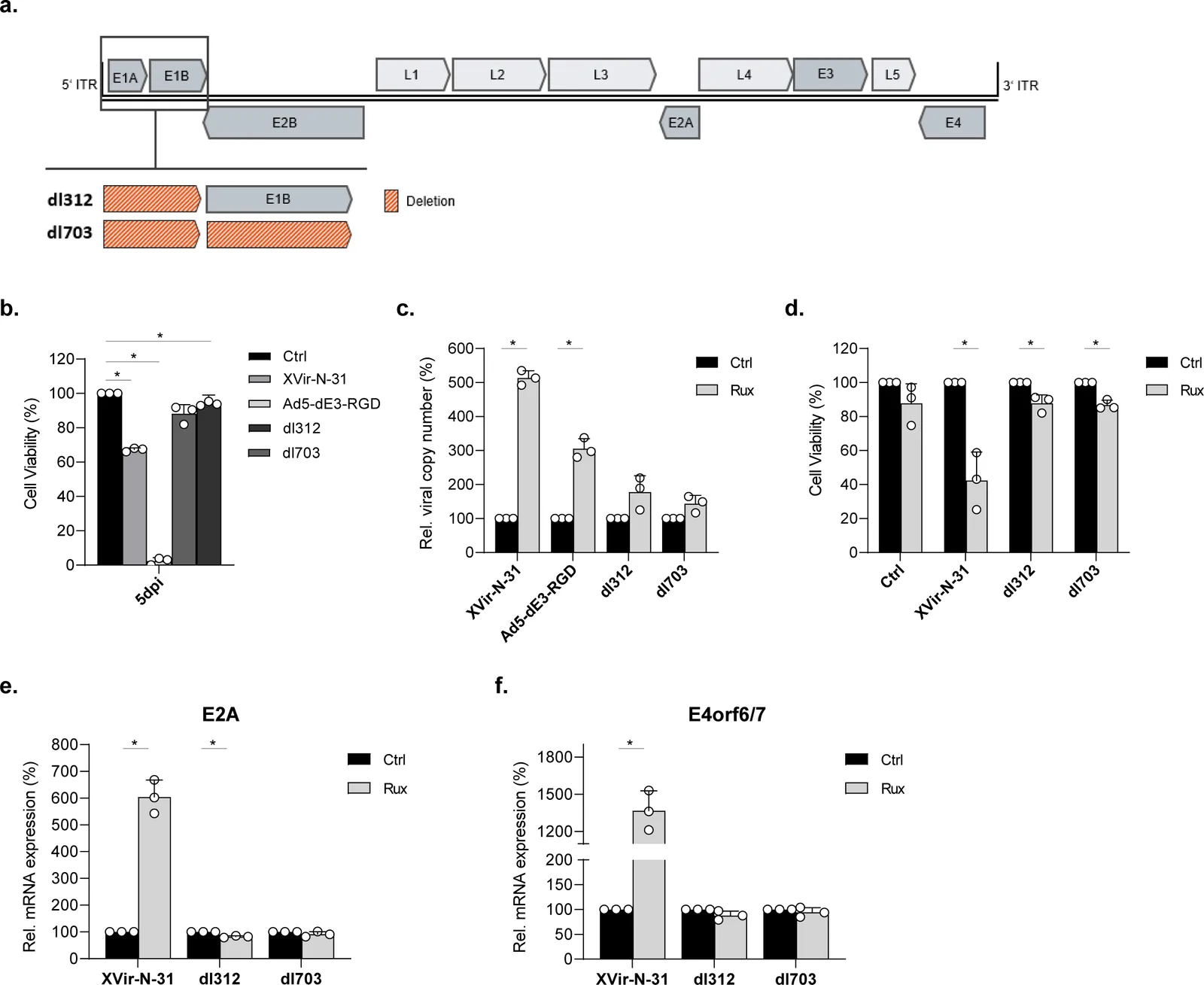
Expression of E1A is a prerequisite for the response to JAK/STAT inhibition. (**a**) Schematic genomic overview of dl312 and dl70-3. (**b–f**). UM-UC-3 cells with or without 24 h pretreatment of Ruxolitinib 10 µM were infected with XVir-N-31, dl312, and dl70-3 (MOI 20). Cell viability was assessed at 5 dpi (**b and d**). Data are shown as the percentage of surviving cells relative to non-infected and untreated control (*n*=3, mean ± SE). Viral genome replication (**c**) was assessed by qPCR to amplify viral fiber DNA. Data are presented as the percentage of relative fiber DNA at 48 hpi, referred to the untreated control (*n* = 3, mean ± SE). Viral gene expression (**e and f**) was analyzed at 24 hpi. Data are represented as the percentage of relative RNA expression at 24 hpi, referred to the untreated control (*n* = 3, mean ± SE). Data were analyzed by using multiple *t*-tests corrected for multiple comparisons by the Holm-Sidak method (*, *P* ≤ 0.05).

E1A is not only the first expressed adenoviral protein but also essential for the transactivation of adenoviral gene expression and numerous functional events in the adenoviral life cycle (41). Since published results show a direct effect of IFN stimulation on E1A expression levels in normal lung epithelial cells (12), we wanted to examine in greater detail the putative functional role of E1A in mediating the response to Ruxolitinib. Therefore, we used the adenoviruses dl312, which is deleted in E1A, and dl70-3, which is deleted in E1A and E1B (42, 43) (Fig. 4a). Both dl70-3 and dl312 exhibit severely suppressed replication, which can be partially recovered by using higher MOIs in cancer cells, an effect described as E1A-like replication (41, 44). Both viruses induce only a low level of cell lysis (Fig. 4b). The positive modulation on cell lysis and viral replication by Ruxolitinib was almost completely abolished in both dl70-3 and dl312 (Fig. 4c and d). Ruxolitinib treatment resulted in a 6-fold enhancement of E2A expression and a 14-fold E4orf6/7 expression in XVir-N-31 at 24 hpi, while the transcription level of E2A and E4orf6/7 was unchanged in both dl312 and dl70-3 (Fig. 4e and f). These findings indicate that E1A expression is required for the suppressive effects of JAK/STAT signaling on adenoviral replication.

### The response to Ruxolitinib on viral replication results from enhanced E1B and particularly E4 expression

**Fig 5:**
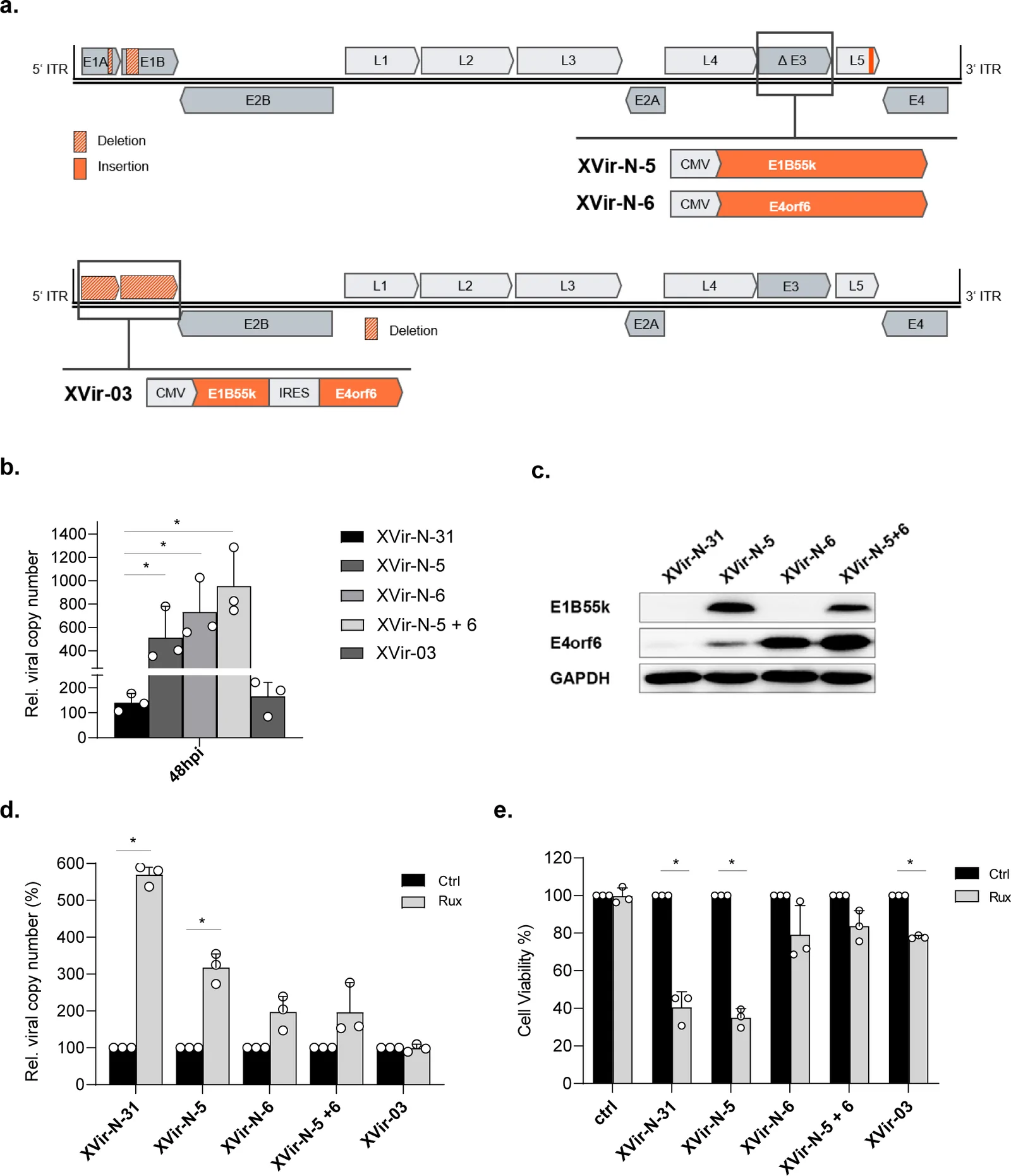
E1B55K and E4orf6 contribute to the effect of JAK/STAT inhibition on adenovirus replication. (**a**) Schematic genomic overview of XVir-N-5, XVir-N-6, and XVir03. (**b and d**) UM-UC-3 cells without (**b**) or with (**d**) 24 h pretreatment of Ruxolitinib 10 µM were infected with XVir-N-31, XVir-N-5, XVir-N-6, and XVir03 (MOI 10) and co-infected with XVir-N-5 (MOI 5) and XVir-N-6 (MOI 5). Replication was assessed by qPCR to amplify viral fiber DNA in UM-UC-3 cells. Data are shown as the absolute value (**b**) or fold change (**d**) of relative fiber DNA at 48 hpi, referred to the untreated control (*n* = 3, mean ± SE). (**c**) Viral protein expression was analyzed by western blotting in UM-UC-3 cells infected with XVir-N-31, XVir-N-5, XVir-N-6, and XVir03 (MOI 10) or co-infected with XVir-N-5 (MOI 5) and XVir-N-6 (MOI 5) for 24 h (*n* = 3, one representative blot is shown). (**e**) UM-UC-3 cells without 24-h pretreatment of Ruxolitinib 10 µM were infected with XVir-N-31, XVir-N-5, XVir-N-6, and XVir03 (MOI 20). Cell viability was assessed at 5 dpi. Data are shown as the percentage of surviving cells relative to the untreated control (*n* = 3, mean ± SE). Data in panel a were analyzed by using an unpaired two-tailed *t*-test, and in panels d and e by using multiple *t*-tests corrected for multiple comparisons by the Holm-Sidak method (*, *P* ≤ 0.05).

Our previous data show that inhibition of JAK signaling results in increased expression levels of E1B55K, E2, and E4. We wanted to address the question of whether upregulation of these early genes is the reason for the observed functional effects. For this purpose, we generated novel adenovirus vectors based on XVir-N-31 by cloning either a CMV-E1B55K or a CMV-E4orf6 expression cassette in the ΔE3 region, resulting in XVir-N-5 and XVir-N-6 (Fig. 5a). In this experiment, we additionally included the virus XVir03, in which the E1 region was replaced by a CMV promoter-driven bicistronic expression cassette comprising E4Orf6 and E1B55K, separated by an internal ribosome entry site (IRES) to enable expression of both genes from a single transcript (45). We first compared the replication of XVir-N-31,-5, -6, and XVir03, as well as a combination of XVir-N-5 and -6. XVir-N-31 and XVir03 resemble each other in replication. Both XVir-N-5 and -6 show a fourfold to sixfold better replication than the other two viruses, with slightly better replication of XVir-N-6 (Fig. 5b). The combination of XVir-N-5 and -6 showed a clear tendency to an even better replication compared to the single viruses. Increased expression levels of the transgenes and confirmation of positive parallel infection of XVir-N-5 and -6 were demonstrated by western blotting against E1B55K and E4orf6 (Fig. 5c).

When combining these viruses with Ruxolitinib, XVir-N-31 showed a sixfold increase in replication, whereas replication of XVir-N-5 was enhanced threefold, and that of XVir-N-6 only twofold, similar to the combination of -5 and -6 (Fig. 5d). XVir03 did not respond to treatment. Interestingly, virus-induced cell lysis was triggered by Ruxolitinib only in the XVir-N-31- and XVir-N-5-infected cells (Fig. 5e), indicating that the E4 region contributes to a higher degree to the positive effect of JAK/STAT inhibition on the viral life cycle than E1B55K.

Together, these findings indicate several aspects: first, they support the observation that the lack of E1A in XVir03, as in dl312 and dl70-3, prevents a response to Ruxolitinib, indicating that E1A is the essential protein in the IFN response. Second, the expression levels of E1B55K and E4orf6 are the dominant downstream elements, responsible for the functional effects induced by Ruxolitinib, with expression of E4 being the dominant gene.

### STAT molecules can contribute in a redundant and phosphorylation-independent mechanism to adenovirus replication

**Fig 6:**
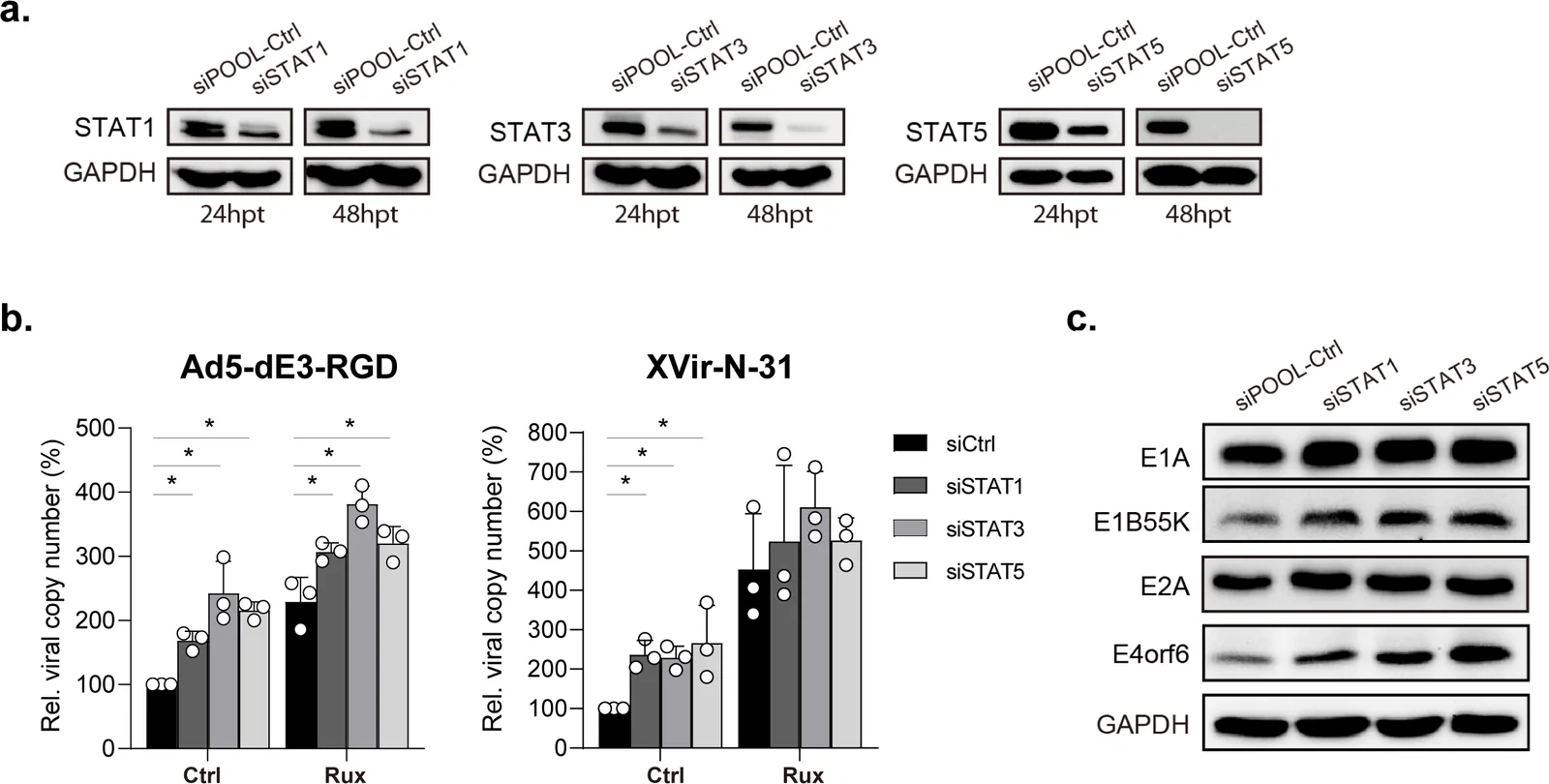
Adenovirus infection and JAK/STAT inhibition effect. JAK/STAT family members and selective knockdown of STAT1, STAT3, or STAT5 increase viral replication. (**a**) siRNA-mediated knockdown of STAT1, 3, and 5 was performed in UM-UC-3 cells by reverse transfection with siPOOL. The knockdown was confirmed by western blotting at 24 hpt and 48 hpt (*n* = 3, one representative blot is shown). (**b**) UM-UC-3 cells transfected with the indicated siPOOL, pretreated without Ruxolitinib 10 µM 6 h later. After 24 h, cells were infected with Ad5-dE3-RGD (MOI 1) or XVir-N-31 (MOI 10), and replication was assessed by qPCR. Data are presented as the fold change of relative fiber DNA referred to the non-transfected control (*n* = 3, mean ± SE). Data were analyzed by using multiple *t*-tests corrected for multiple comparisons by the Holm-Sidak method (*, *P* ≤ 0.05). (**c**) UM-UC-3 cells transfected with the indicated siPOOL, at 24 hpt, were infected with XVir-N-31 (MOI 10). Viral proteins were analyzed by western blotting at 24 hpi (*n* = 3, one representative blot is shown).

The contributions of STAT1, STAT2, and STAT5 to adenovirus replication have been reported previously (12, 19, 26–28, 46). Thus, we used siRNAs (siTool) to suppress the expression of these 3 STAT isoforms and demonstrated efficient ablation of all three STATs over 2 days (Fig. 6a). We could also show that the replication of both Ad-dE3-RGD and XVir-N-31 was upregulated twofold to threefold by all siRNAs. However, treatment with Ruxolitinib still had the same effect on replication as the siRNA control (Fig. 6b). Since STAT2 and -5 are not present in their phosphorylated form in the UM-UC-3 cells, as shown in Fig. 1b, this mechanism does not depend strictly on STAT phosphorylation. We also confirmed our observation that early genes are expressed at a higher level when combining JAK or STAT inhibitors and adenovirus by showing that the siRNAs used caused the same effect (Fig. 6c).

### Trapping of STAT proteins, using a STAT consensus sequence in the viral genome, improves oncolytic efficacy

**Fig 7:**
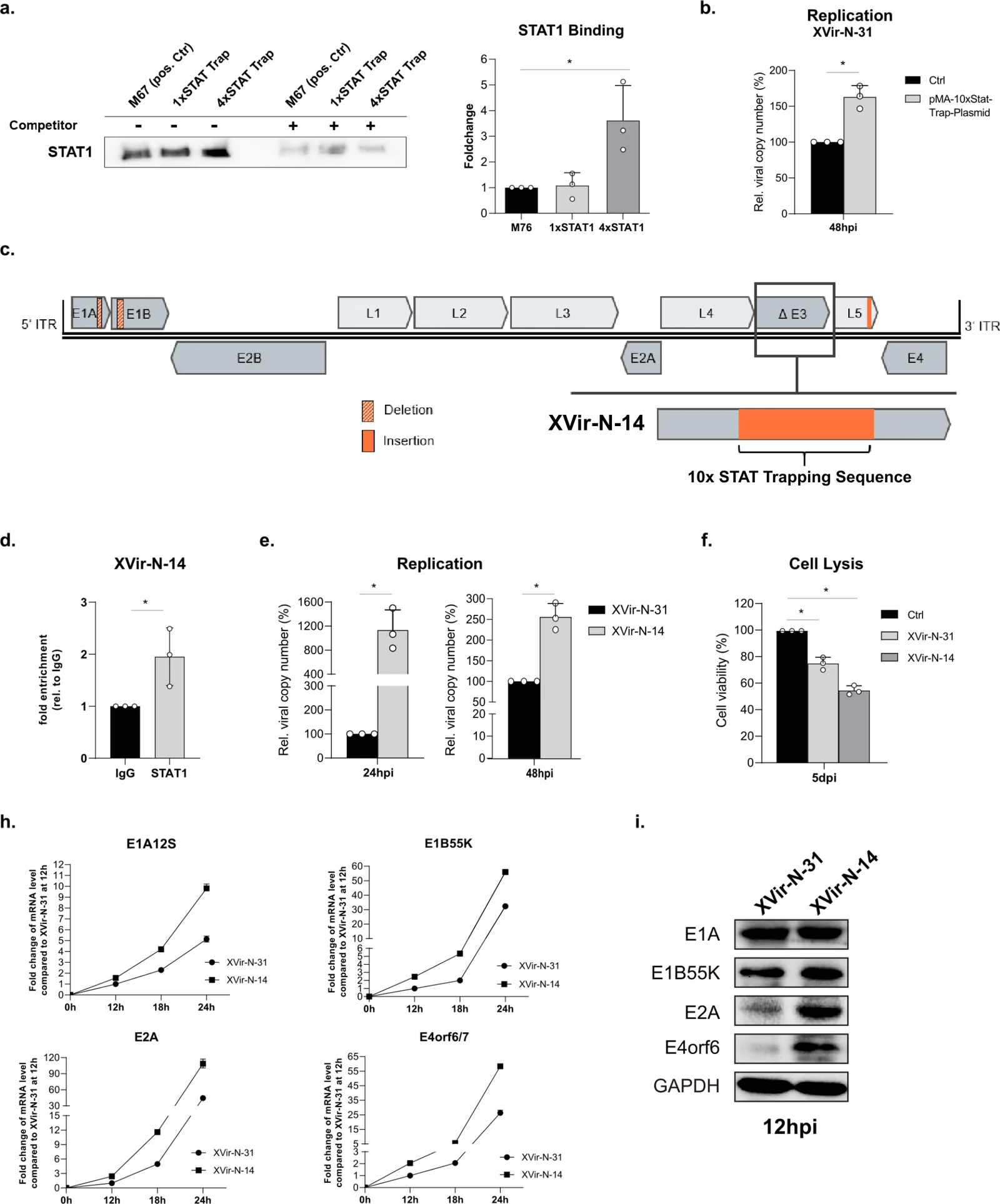
Trapping STAT proteins improves replication. (**a**) DNA pull-down was performed by adding annealed 5′-biotinylated DNA oligonucleotides (M76, 1xSTAT, and 4xSTAT) without competitor to UM-UC-3 cells. Pull-down of protein–DNA-binding complexes was performed using streptavidin-conjugated resins and analyzed by western blotting (*n* = 3, one representative blot is shown). (**b**) UM-UC-3 cells were infected with XVir-N-31 (MOI 10) at 24 h post-transfection of pMA-10x STAT Trap (10 STAT-binding site repetitions). Viral genome replication was assessed by qPCR. Data are presented as fold changes relative to the untreated control (*n* = 3, mean ± SE). (**c**) Schematic genomic overview of XVir-N-14. (**d**) Chromatin Immunoprecipitation was performed by infecting UM-UC-3 cells with XVir-N-14 (MOI 50) and cross-linking at 18 hpi. Cross-linked chromatin was sheared by sonication, and immunoprecipitation was performed using STAT1 antibody and IgG antibody as a negative control. Precipitated DNA was quantified by qPCR (*n* = 3, mean ± SE). (**e–i**) UM-UC-3 cells were infected with the indicated viruses at an MOI of 10. Viral genome was assessed by qPCR to amplify viral fiber DNA at 24 hpi (**e**). Data are presented as fold changes relative to the untreated control (*n* = 3, mean ± SE). Viral gene expression was analyzed at 12, 18, and 24 hpi. Data are presented as fold changes relative to the 12 h XVir-N-31 value as the control (**h**) (*n* = 3, mean ± SE). Protein expression was analyzed by western blotting at 12 hpi (**i**). (*n* = 3, one representative blot is shown). Data in panels a, b, d, and f were analyzed by performing an unpaired two-tailed *t*-test (*, *P* ≤ 0.05).

Translating these results into the design of a novel oncolytic virus, we considered including a DNA-binding consensus sequence for STAT isoforms into the viral backbone, which should sequester available STAT proteins. The strategy to sequester a transcription factor has been applied by our group before for trapping free available E2F (36). Thus, we designed the STAT-binding consensus sequence 5′-CATTTCCAGGAAATC-3′ (STAT-TRAP) based on published consensus sequences for STAT binding (47–49). Binding of this oligonucleotide to STAT1 was tested by performing a DNA pulldown. As a control, we included a previously published sequence (M67) recognized by STAT1 (37). We could show that our newly designed sequence binds STAT1 as efficiently as the control and that a 4× multimer binds with a threefold higher affinity to STAT1 (Fig. 7a). Next, we cloned a 10× repetition of this sequence into the pMA reporter plasmid, and transfection of this plasmid 24 h before infection of XVir-N-31 resulted in a 1.5-fold increase in replication (Fig. 7b).

Based on these results, a novel oncolytic virus was designed by cloning a 10× multimer of the STAT-TRAP sequence into the deleted E3 region of XVir-N-31, resulting in the novel virus XVir-N-14 (Fig. 7c). When infecting cells, immunoprecipitation of STAT1 showed specific binding to this nucleotide (Fig. 7d). This virus showed 11.3-fold higher replication after 24 h and 2.5-fold higher replication after 48 h compared to XVir-N-31 (Fig. 7e). Cell viability was decreased by 25% when XVir-N-31 was used; this effect was improved by a factor of 1.8 to 45% when XVir-N-14 was used (Fig. 7f). Compared to XVir-N-31, early gene expression is induced already at 12 hpi, and protein expression levels of E2A and E4orf6 are significantly upregulated (Fig. 7g and h). Similar to the use of JAK/STAT inhibitors, we could not observe an increase in E1A expression. Together, we demonstrate that inhibition of JAK/STAT signaling results in improved replication and an almost twofold increase in cell lysis, using derivatives of adenovirus type 5. This effect requires E1A as an essential factor, which has been described as a crucial transactivator of E2 and E4 transcription.

## DISCUSSION

In this study, we aimed to gain a better molecular mechanistic understanding of the IFN-mediated antiviral defense mechanism against adenoviral infection to further improve the efficacy of oncolytic virotherapy. Our results allowed us to develop a novel concept to optimize replication of the oncolytic adenovirus XVir-N-31, resulting in improved cell lysis.

Similar to findings with wild-type adenovirus type 5 (wtAd5), infection with the wtAd5 derivative Ad5-dE3-RGD and also the oncolytic, E1A13S-deleted virus XVir-N-31 resulted in the upregulation of JAK-1, STAT-1, and STAT-3 phosphorylation levels in a temporally defined kinetics, demonstrating that the host cells’ IFN response is an early antiviral event (50). Treatment with IFN-α or IFN-γ can inhibit adenoviral replication in primary bronchial epithelial cells and, in this study, in UM-UC-3 cells (12). Interestingly, it was absent in the lung adenocarcinoma cell line A549, although the replication of a vesicular stomatitis virus remained sensitive to IFN treatment in these cells (51). Thus, identification of molecular markers that regulate these processes could be valuable to predict IFN-dependent replication and thus therapy success of oncolytic virotherapy.

We show that inhibition of JAK1/2 and STAT3/5 improved the potency of oncolytic adenoviral therapy in bladder cancer cells. The combination of JAK inhibitors with increasing MOIs of both Ad5-dE3-RGD and XVir-N-31 resulted in a synergistic cytotoxic effect and enhanced replication in several bladder cancer cell lines. Prior studies also reported that suppression of JAK1/2 induced adenovirus replication in lung-, breast-, colorectal-, pancreatic-, and ovarian cancer cell lines (28). Also, downstream signal transducers such as STAT1, STAT2, and STAT5 have previously been shown to modulate adenovirus replication (12, 19, 26–28, 46). We demonstrated that multiple members of the STAT family can inhibit adenoviral replication in a redundant, phosphorylation-independent manner, highlighting the critical role of STAT proteins in cellular antiviral defense. This suggests that the strategy of suppressing IFN signaling is beneficial for supporting the therapeutic efficacy of oncolytic adenovirus therapy. Our results also reveal that the effect of JAK1/2 inhibition is not dependent on the pretreatment time but is a direct and immediate event.

Previously, crosstalk between IFN signaling and the E2F/Rb axis has been reported as relevant to IFN-mediated downregulation of E1A, thereby repressing adenoviral replication (12, 13). IFN stimulation was shown to reduce E1A enhancer activity by blocking GABP binding and by enhancing the association of E2F repressor proteins p107 and Rb, leading to persistent infection in primary bronchial epithelial cells. We demonstrate that in bladder cancer cells, adenoviral replication was improved after JAK1/2 inhibition, regardless of the cellular Rb status. However, although E1A expression levels were not modulated by JAK/STAT inhibitors in our observations, its expression is essential in mediating the observed upregulation of early viral genes. The impact of JAK1/2 inhibitors was completely abolished when using the E1/E1A-deleted adenoviruses dl703 and dl312, highlighting the functional importance of E1A. Previous analyses in A549 cells using adenoviruses with deletions in the individual early regions also emphasized the role of E1A in this context (51). Furthermore, we demonstrated that the enhanced expression of E4orf6 and, to a lesser extent, E1B55K constitutes the direct downstream molecular mechanism driving this regulatory phenomenon. This is supported by the observation that viruses simultaneously overexpressing both viral genes exhibited either an abolished or a significantly attenuated response to IFN inhibition (Fig. 5).

It is well established that E1A transactivates the expression of early genes within the adenoviral genome (41, 52–55). Specifically, E2 transactivation is mediated by E1A binding to the retinoblastoma (Rb)-E2F protein complex; this disrupts the complex and releases active E2F, which subsequently interacts with the two inverted E2F binding sites in the E2 promoter. Additionally, all early promoters are regulated via acetylation, a process modulated by the interaction of E1A with the p300**/**CBP complex. E1A is also essential for inducing the proteolytic cleavage of p120E4F1 into the p50E4F1 isoform, which initiates E4 transcription (56).

Adenoviruses harboring a deletion of the E1A region are largely replication-deficient, whereas XVir-N-31, which lacks only the E1A13S isoform, retains replication competence, albeit at a lower efficiency than wild-type adenovirus (30, 44). The E1A13S isoform primarily functions as a potent transactivator of viral early gene transcription, whereas the E1A12S isoform predominantly modulates host cell pathways through interactions with cellular factors, including p300/CBP and the pocket proteins RB1, p107, and p130 (52). Importantly, E1A12S has also been shown to interact directly with STAT1 through a p300/CBP-independent binding site (18). Our findings extend these observations by demonstrating that pharmacological inhibition or reduced availability of STAT proteins in the presence of E1A12S suppresses adenoviral replication.

Thus, we investigated whether a targeted approach could be engineered to sequester STAT proteins while preserving the host’s systemic immune response, which remains crucial for successful oncolytic therapy. Various therapeutic modalities, including siRNA, shRNA, antisense oligonucleotides (ASOs), and oligodeoxynucleotide (ODN) decoys, have been developed and evaluated for cancer therapy; however, none of these approaches has received regulatory approval, largely due to severe adverse effects (57–59). Inspired by a study from He et al. (60), in which E2F was successfully sequestered by introducing a plasmid containing a multimerized E2F consensus binding sequence into cells, we previously adapted this principle by cloning a cassette with multimerized E2F-binding sites from the E2 promoter into the E3 region of wild-type adenovirus (36). To adapt this molecular mechanism into a therapeutic platform, we developed an innovative paradigm for the design of oncolytic virotherapy by introducing a multimer of a STAT consensus sequence into the viral genome, trapping nuclear STAT molecules at this site. We successfully demonstrated STAT1 binding to this sequence within the viral genome and confirmed that this integrated approach improved replication and oncolysis of XVir-N-14 compared to the parental virus. Mechanistically, our findings allowed us to establish a refined approach to optimize the replication of the parental virus, leading to enhanced cell lysis.

**Fig 8:**
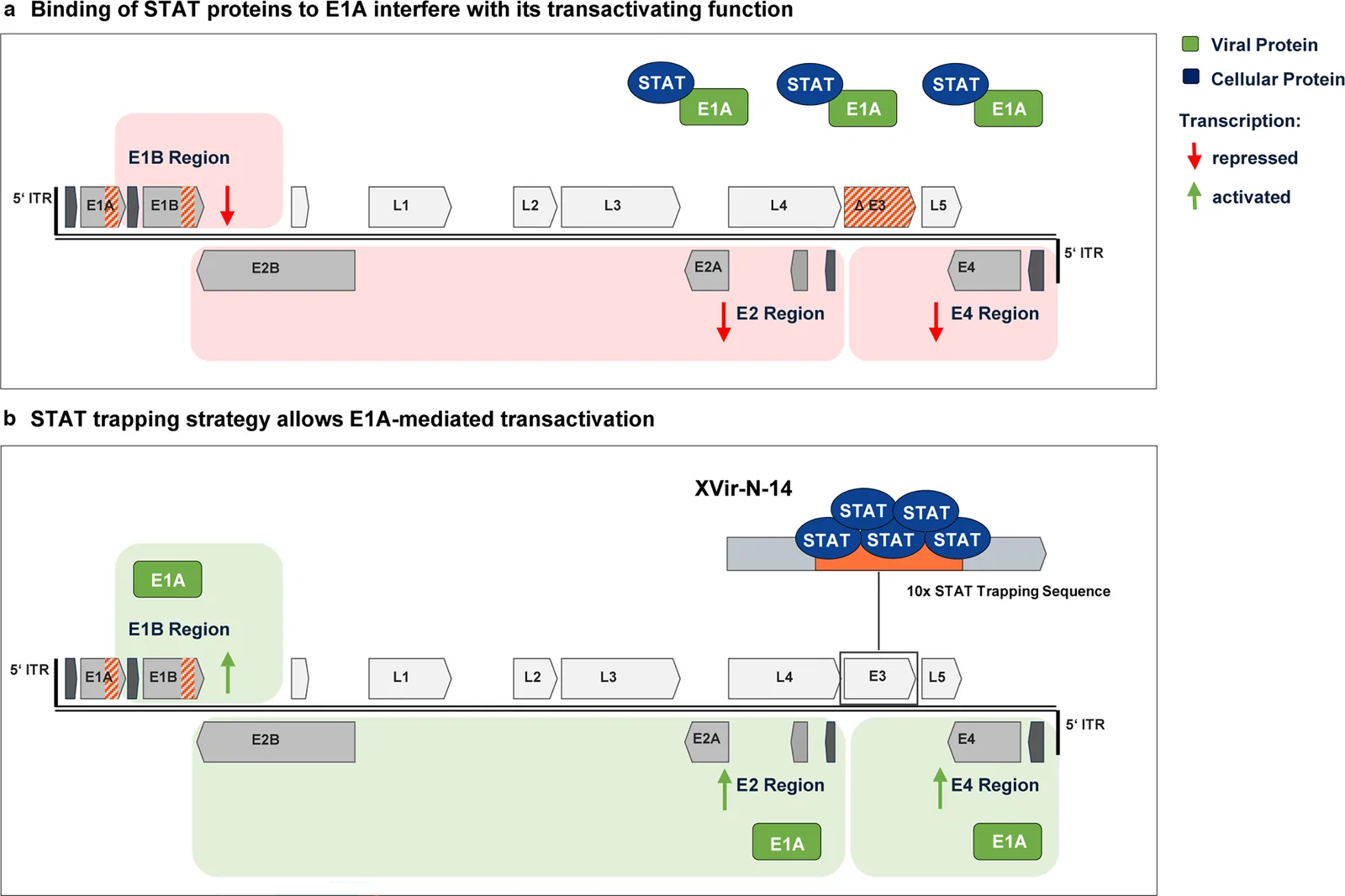
Proposed model for the molecular mechanism by which STAT proteins regulate E1A-mediated transactivation of early viral gene expression. (**a**) In cancer cells, the formation of E1A–STAT protein complexes partially restricts the transactivation activity of E1A, thereby reducing transcription of early viral genes. (**b**) Introduction of a STAT decoy sequence into the adenoviral genome sequesters STAT proteins, thereby preventing or reducing E1A–STAT complex formation. Consequently, E1A remains available to transactivate early viral gene expression, resulting in enhanced transcription of early viral genes.

These data support a model in which the transactivation activity of E1A at viral early promoters is partially impaired through the formation of E1A–STAT protein complexes. We propose that these complexes impose steric constraints that interfere with the recruitment of the transcriptional machinery and thereby reduce E1A-mediated transactivation of early viral gene expression (Fig. 8a).

Insertion of a STAT-specific decoy sequence into the viral genome should prevent or substantially reduce the formation of E1A–STAT complexes. Consequently, a greater proportion of E1A would remain available to activate early viral transcription (Fig. 8b).

However, the positive effect on replication of XVir-N-14 was less pronounced than that achieved with pharmacological JAK/STAT inhibitors. This discrepancy likely arises because the success of this molecular trapping mechanism depends on the cellular abundance of STAT molecules balanced against the offered available STAT-binding sites, which are strictly limited by the copy number of viral genomes in the cell. Since our data indicate that the most critical effect of the IFN response occurs at early stages of the viral life cycle, the relatively low number of viral genomes at the point of infection presents a clear limitation for this concept during the initial phases of replication. Nevertheless, our methodology offers a novel direction to avoid toxicities and enhance the oncolytic effect when using conditional therapeutic viruses and represents an additional approach to interfere with the JAK/STAT signaling pathway beyond the already successfully used shRNAs against STAT3 in M4 or expression of the JAK inhibitor in oAd-SOCS3 (28, 46, 61). Furthermore, the approach of trapping STAT molecules could be extended to other transcription factors of the cellular antiviral system to generate improved oncolytic adenoviruses. The IFN responsiveness observed in E1A-expressing adenoviruses is highly relevant beyond XVir-N-31, because many clinically established oncolytic adenoviral vectors retain E1A-dependent replication programs, including DNX-2401 (Tasadenoturev; Delta-24-RGD), ONYX-015 (dl1520), Cretostimogene Grenadenorepvec (CG0070), and LOAd703 (Delolimogene Mupadenorepvec) (62). Thus, modulation of IFN/STAT-mediated restriction may represent a broadly applicable strategy to improve the efficacy of replication-competent adenoviral therapeutics.

## Data Availability

All data that support the findings of this study are provided in this article. Data beyond the results shown are available from the corresponding author upon reasonable request.

## SUPPLEMENTAL MATERIAL

Figure S1 jvi.01068-26-s0001.tif Bladder cancer cell lines were pretreated with indicated concentrations of JAK1/2 inhibitors for 24 h and then infected with indicated MOIs of Ad5-dE3-RGD or XVir-N-31.

Figure S2 jvi.01068-26-s0002.tif UM-UC-3 cells were pre-treated with ruxolitinib for various times and then infected with XVir-N-31.

Supplemental legends jvi.01068-26-s0003.docx Legends for Fig. S1 and S2.

ASM does not own the copyrights to Supplemental Material that may be linked to, or accessed through, an article. The authors have granted ASM a non-exclusive, world-wide license to publish the Supplemental Material files. Please contact the corresponding author directly for reuse.
