# Supplementary material for: Integration of a multimeric STAT consensus site into the adenoviral genome to interfere with IFN signaling represents a novel concept to improve oncolytic virotherapy
Source: J Virol. 2026 Aug 10;100(9):e01068-26. doi: 10.1128/jvi.01068-26 (PMC13595963; doi:10.1128/jvi.01068-26)
Supplement: Supplementary file 1 [file jvi.01068-26-s0003.docx]

**Figure Legends to supplement**

**Supplement Figure 1:**

**(a-j)** Bladder cancer cell lines (UM-UC-3, 5637, T24, T24shRb) were pretreated with indicated concentration of JAK1/2 inhibitors (Ruxolitinib (Rux), Itacitabine (Ita), Napabucasin (Nap)) for 24 h and then infected with indicated MOIs of Ad5-dE3-RGD (WT) or XVir-N-31 (XVir). Cell viability was assessed at 5 dpi (n = 3, mean ± SE). Synergy Scores between JAK1/2 inhibitors and viruses across different cell lines were calculated using the ZIP model.

**Supplement Figure 2:**

**(a-d)** UM-UC-3 cells were pre-treated with Ruxolitinib (10 μM) for 0 h, 12 h, and 24 h, and then infected with XVir-N-31 (MOI 10). To analyze viral replication, cells were collected at 24 hpi, and viral fiber DNA was amplified by qPCR. Data are normalized to the 4 h fiber DNA value and shown relative to the untreated control (**c,e**) (n=3, mean ± SE). Cell viability was measured by fixing the infected cells 5 dpi and performing an SRB assay (**d-f**) (n=3, mean ± SE). Data are analyzed by performing an unpaired two-tailed t-test (*, P ≤ 0.05).
